# Flexible and Electroactive Textile Actuator Enabled by PEDOT:PSS/MOF-Derivative Electrode Ink

**DOI:** 10.3389/fbioe.2020.00212

**Published:** 2020-03-19

**Authors:** Yanxiao Wu, Ying Yang, Cheng Li, Yunbo Li, Wei Chen

**Affiliations:** ^1^School of Materials Science and Engineering, Shanghai University, Shanghai, China; ^2^i-Lab, Suzhou Institute of Nano-Tech and Nano-Bionics, Chinese Academy of Sciences, Suzhou, China; ^3^Nanchang Research Institute, Suzhou Institute of Nano-Tech and Nano-Bionics, Chinese Academy of Sciences, Nanchang, China; ^4^Research Centre for Smart Wearable Technology, Institute of Textiles and Clothing, The Hong Kong Polytechnic University, Kowloon, Hong Kong; ^5^Shenzhen Research Institute, The Hong Kong Polytechnic University, Shenzhen, China

**Keywords:** PEDOT:PSS, MOF derivative, fabric electrolyte layer, i-EAP actuator, textile actuator

## Abstract

Smart fabrics that integrate electronic devices with textiles are emerging as potential candidate for apparel and electronics industries. Soft actuators based on conducting polymers are promising for smart fabrics because of light weight, flexibility, and large deformation under low voltage. However, due to the distinct characteristics of textile and electronic components, the connection between textiles and electronic devices still keeps a challenge in development of smart fabrics. Here, we report an new strategy to prepare a flexible and electroactive textile actuator. The fabric electrolyte was directly coated with an electrode ink, which is composed of Poly(3,4-ethylenedioxythiophene):poly(styrene sulfonic acid) doped with carbonized carbon nanotubes wired zeolite imidazolate framework-8 composite. A pre-treatment of the fabric was made by soaking hydrophobic poly(vinylidene fluoride-co-hexafluoropropylene) to increase the ionic conductivity (6.72 mS cm^–1^) and prevent the electrode ink from penetrating through the fabric. It was found that the textile actuator could work in air stably under a low voltage of 3 V and operate at frequencies from 0.1 to 10 Hz with large strain difference (0.28% at 0.1 Hz), fast strain rate (2.8% s^–1^ at 10 Hz) and good blocking force (0.62 mN at 0.1 Hz). The key to high performance originates from high ionic conductivity of fabric electrolyte and large specific surface area, good mechanical properties of the metal-organic framework derivative-based composite electrodes, which present insights into preparing other smart fabrics such as textiles sensors, flexible displays, and textile energy storage devices.

## Introduction

Since the prehistoric times, humans have been using textiles, which is important and inseparable part of human life (Kaushik et al., 2015). With the development of electronics and textiles technology, textiles have broken through the scope of insulation and beautification. Textiles with more functions such as display, sensing, actuation, energy collection, and storage have received widespread attention (Kaushik et al., 2015; Liu M. et al., 2017; Maziz et al., 2017; Zhang et al., 2018). However, since textiles are soft and compliant while electronic devices are rigid, the differences in structure and performance between the two cause the combination of electronic components and textiles to be an obstacle in the path of developing electronic textiles. In general, the preparation of electronic fabrics can be divided into two categories: one is to embed electronic components on conventional fabrics; other one is to integrate electronic devices directly on the fabric (Kaushik et al., 2015; Zahid et al., 2017; Shi et al., 2019).

The flexible intelligent actuator as a very important part of the electronic fabric can directly convert the external energy such as electricity, light, heat and humidity into the mechanical deformation individually, without need of a cumbersome energy conversion device (Dai et al., 2013; Hu et al., 2017; Lu et al., 2018; Wang et al., 2019). Electroactive polymers (EAPs) have been widely used in the field of actuators due to their special electrical and mechanical properties. In particular, ionic electroactive polymers (i-EAPs) allow ions to migrate and move inside, which achieve volume and size changes by expansion, contraction, etc., thereby completing the conversion between mechanical and electric energy (Bhandari et al., 2012; Terasawa and Asaka, 2015; Yan et al., 2017). Among several types of i-EAPs, poly(3,4-ethylenedioxythiophene):poly(styrene sulfonic acid) (PEDOT:PSS) is a good candidate for electrode materials of flexible actuators due to its biocompatibility, fast reversible redox process and light weight. PEDOT:PSS-based actuators can generate large deflection displacements at low voltages (typically <3 V) and have long cycle life (>10^5^ cycles) as well as high driven efficiency (Smela, 2003; Wang et al., 2017; Park et al., 2018). However, in general, combination of electrode film and electrolyte layer is by means of hot pressing, which leads to poor the interface bonding, and affect cycle stability of actuators (Madden et al., 2007; Bar-Cohen et al., 2015; Melling et al., 2019). Some new processes have also been proposed for the fabrication of actuator devices. For instance, Põldsalu et al. (2017) used an inkjet printing method to prepare a PEDOT:PSS-activated carbon linear actuator that can operate in an organic solvent. Aiva Simaite et al. hydrophilically treated the surface of the hydrophobic poly(vinylidene fluoride-co-hexafluoropropylene) PVDF-HFP intermediate layer so that the electrode solution can be directly applied to the intermediate layer to form a film (Bar-Cohen et al., 2015).

In order to further improve the actuating performance of the i-EAP-based actuator device, many kinds of materials [such as multi-walled carbon nanotubes (MWCNTs) (Wang et al., 2017), polyethylene oxide (PEO) (Rohtlaid et al., 2019), zirconium(IV) phosphate (ZrP) (Inamuddin et al., 2015), etc.] are integrated into polymer matrix to prepare a composite electrode material with superior performance. Zeolitic imidazolate framework-8 (ZIF-8) and its derivatives are widely used in electrode materials or additives due to their large specific surface area (SSA) and hierarchical porous structure. Samuel et al. (2018) prepared an electrode film for a lithium ion battery for storing lithium ions by depositing ZIF-8 onto electrospun polyacrylonitrile-2-methyl imidazolate (PAN-2MI) nanofibers and then carbonizing Chao Lu et al. used a polyhedron prepared by direct pyrolysis of CNTs/ZIF-8 composite as an electrode to obtain a flexible supercapacitor with large specific capacitance and high energy density (Lu et al., 2017). Based on previous studies, it is clearly found that ZIF-8 and its derivatives are promising electrode materials of actuators with large ion accommodation.

Here, we synthesized the SWCNT wired ZIF-8 structure by *in situ* growth method, and prepared the conductive ink by compositing the carbonized product with PEDOT:PSS. In addition, we prepared the electrolyte intermediate layer by immersing the polyester fiber fabric in PVDF-HFP solution, which not only increased the ionic conductivity of the fabric (6.724 mS cm^–1^) but also prevented the electrode ink passing through the fabric from causing a short circuit. Then, the surface of the fabric is subjected to hydrophilization treatment by a plasma etching technique, so that the electrode ink can be directly formed on surface of the fabric. This method endows a good interface contact between electrode layer and electrolyte layer of the device. The prepared textile actuator exhibits a large strain difference (0.28%), a strain rate (2.8% s^–1^), and a high blocking force (0.62 mN at 0.1 Hz), which shows great application prospects in the field of auxiliary medical and wearable electronics.

## Materials and Methods

### Materials

Polyester fabric was purchased from Wenzhou Chaofan Textiles Co., Ltd., SWCNT wired ZIF-8 was synthesized according to previous literature (Lu et al., 2017). SWCNT-COOH was brought from Nanjing XFNANO Materials Tech Co., Ltd., Anhydrous methanol, Zn(NO_3_)_2_∙6H_2_O and N,N-dimethylformamide (DMF) were obtained from Shanghai chemical reagent Co., Ltd., 2-methylimidazole was purchased from Aladdin Reagent. PEDOT:PSS pellets and PVDF-HFP were obtained from Sigma-Aldrich. Ionic liquid (EMIBF_4_) was bought from Shanghai Cheng Jie Chemical Co., Ltd.

### Synthesis and Fabrication

#### Synthesis and Carbonization of SWCNT Wired ZIF-8

Herein, we used the classical precipitation synthesis route for SWCNT wired ZIF-8 (Lu et al., 2017). SWCNT (40 mg) was dispersed in anhydrous methanol for 0.5 h with ultrasonication in an ice water bath. And then Zn(NO_3_)_2_∙6H_2_O (700 mg) was added to the solution and stirred for 1 h to form a homogeneous solution A. Subsequently, 2-methylimidazole (1050 mg) was dissolved in anhydrous methanol to form a clear solution B, which was added dropwise to the solution A with stirring vigorously. After stirring for 1 h, the solution was allowed to stand without any disturbance for 24 h. The black precipitate obtained by centrifugation was collected and dried under vacuum at 60°C after washed five times with methanol. The dried powder was uniformly dispersed in a crucible and placed in a tube furnace. The crucible was heated to 200°C at a rate of 2°C min^–1^ and kept for next 2 h to remove the organic phase from the material. And then heated to 800°C at 5°C min^–1^ and held for 3 h to ensure that the precursor material was completely carbonized.

#### Preparation of Electrode Ink

The products obtained by pyrolysis were taken from 0 mg, 20 mg, 30 mg, and 40 mg, respectively, and added to deionized water (10 g), then sonicated for 1 h in an ice water bath. PEDOT:PSS pellets (0.2 g) were added to the dispersion, and the mixture was stirred overnight to obtain a mixture dispersion. The electrode inks prepared by adding 0 mg, 20 mg, 30 mg and 40 mg of carbonized MOF were named as PEDOT:PSS, 10% CCZ8/P.P, 15% CCZ8/P.P, and 20% CCZ8/P.P.

#### Preparation of Fabric Layer

**FIGURE 1:**
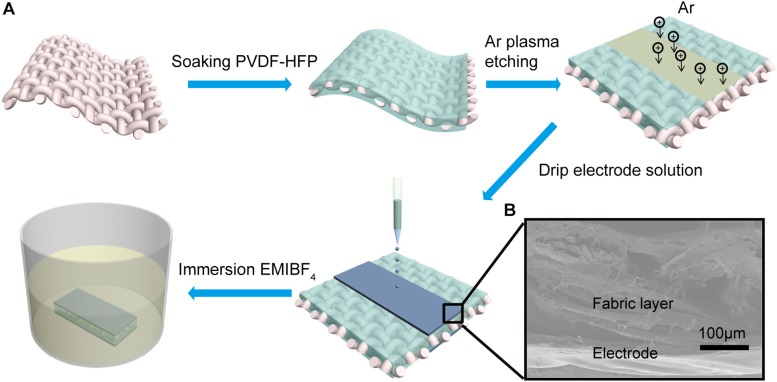
**(A)** Schematic diagram of the textile actuator assembly and configuration. **(B)** SEM cross-sectional image of the fabric driver shows good interfacial adhesion.

Gel polymer electrolyte solution was obtained by dissolving PVDF-HFP (5 g) in DMF at room temperature. As illustrated in Figure 1, polyester fabrics were immersed into the solution for different times after tailored with certain size. These were subjected to plasma etching in N_2_ atmosphere to obtain a surface hydrophilic fabric layer utilizing magnetron sputtering coating system JGP500 (Beijing Lako Roya Technical Co., Ltd.).

#### Fabrication of the Textile Actuators

Figure 1A shows the process of a textile actuator assembly and configuration. The prepared electrode ink was directly applied dropwise to intermediate layer etching region. Then the fabric was placed on a heating platform to bake at 40°C for 2 h. Repeated this on the other side. The fabric was then tailored (0.5 cm × 3 cm size) and immersed in EMIBF_4_ for 2 h to allow the fabric layer absorbing sufficient ionic liquid. Finally, the ionic liquid on surface of the textile actuator was wiped with absorbent paper. It can be seen that the electrode layers make good interlayer adhesions with the fabric electrolyte layer (Figure 1B), causing faster ion migration and good mechanical properties of textile actuator.

### Characterization

All the electrochemical characterizations were recorded by a Huachen electrochemical workstation CHI 660C. Scanning electron microscope (SEM) images were recorded by Hitachi S-4800 under 10 kV, 10 μA. The tensile properties were tested by utilizing a mechanical tester (AGS-X, Shimadzu) at the tensile speed of 1 mm/min. The electrical conductivity was tested by the multifunction digital four-probe tester (ST-2258C). Raman spectra was conducted with a LabRAM HR800 from JY Horiba. X-ray diffraction (XRD) analysis was measured by Philips X’Pert PRO diffractometer with nickel-filtered Cu Kα radiation. Nitrogen sorption analysis was analyzed by Micromeritics ASAP 2020 instrument using Brunauer–Emmett–Teller (BET) method. Actuation displacement was recorded by laser locator (Keyence, LK-G80). The blocking force was measured utilizing a load cell (JZ-101, XINHANG).

## Results

### Electrode Material Characterization

**FIGURE 2:**
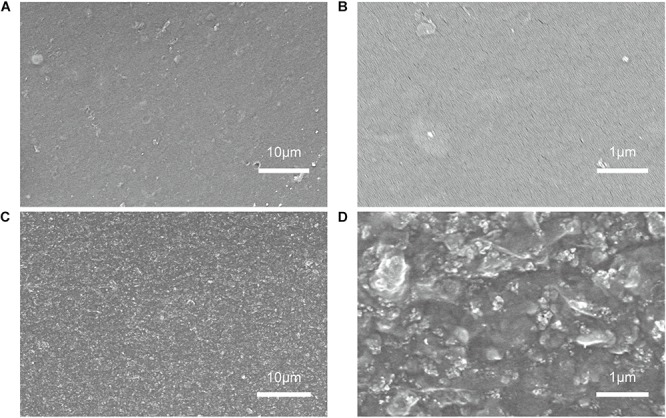
The surface morphology of the electrode film at different magnification. **(A,B)** The PEDOT:PSS electrode film; **(C,D)** The 20% CCZ8/P.P electrode film.

We used electrode ink to prepare self-standing electrode films by casting to facilitate the characterization. In order to investigate the effect of the addition of CCZ8 on the micro-structure of PEDOT:PSS electrode films, SEM images were presented in Figure 2. Figures 2A,B revealed that the surface topography of a pristine PEDOT:PSS electrode film was smooth and dense. While the surface morphology of the electrode film to which CCZ8 was added became significantly rougher, and the distribution of CCZ8 was homogenous in the PEDOT:PSS matrix (Figures 2C,D). In addition, PEDOT:PSS dispersion with fluidity was filled in the pores of the polyhedral skeleton.

**FIGURE 3:**
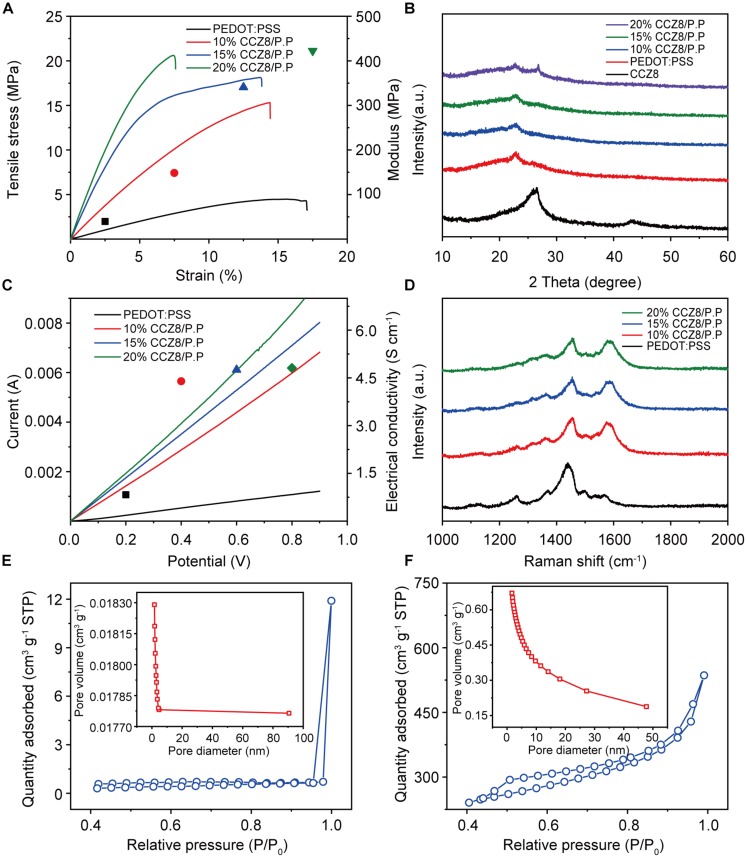
Properties and chemical analysis of electrode films **(A)** Stress-strain curves of electrode films. **(B)** XRD patterns of electrode films. **(C)** I-V curves of electrode films. **(D)** Raman spectra of electrode films. **(E)** N_2_ adsorption/desorption isotherms and pore size distribution (inset) of 20% CCZ8/P.P electrode films. **(F)** N_2_ adsorption/desorption isotherms and pore size distribution (inset) of CCZ8 powder.

To explain the influence of adding CCZ8 on the mechanical properties of the electrode films, the tensile properties were tested. Tensile modulus, tensile strength, and elongation at break of PEDOT: PSS electrode films with 0–20% CCZ8 added were calculated from the stress–strain curves (Figure 3A). The maximum fracture stress and strain elongation of the pristine PEDOT:PSS film were 4.49 MPa and 17.04%, respectively, while the stress and strain of the 20% CCZ8/P.P film reached 20.65 MPa and 7.55%. The Young’s modulus of the electrode materials are summarized in Table 1. As the amount of CCZ8 increased, the Young’s modulus increased from 39.68 MPa of the pristine PEDOT:PSS film to 422.48 MPa of 20% CCZ8/P.P. Actually, it is still lower than many PEDOT: PSS electrodes prepared by commercial dispersions (e.g., Clevios PH1000). These results indicate that the stiffness of the electrode films gradually increased with the addition of carbon polyhedral particles. Figure 3C shows that the conductivity of the PEDOT:PSS electrode film with no addition is only 0.83 S cm^–1^, while the conductivity of 20% CCZ8/P.P film (4.81 S cm^–1^) reaches 5.8 times that of the pristine PEDOT:PSS film. The electrical conductivity of the electrode films increase remarkably with the amount of the additive increases, because the added CCZ8 forms a conductive skeleton in the electrode film, which is favorable for the movement and transmission of electrons.

**TABLE 1:** Specific surface areas, pore size, total pore volumes, electrical conductivity and Young’s modulus of materials used in electrodes.

**Materials**	**Thickness (μm)**	**SSA (m^2^ g^–1^)**	**Pore size (nm)**	**Total pore volume (cm^3^ g^–1^)**	**Electrical conductivity (S cm^–1^)**	**Young’s modulus (MPa)**
10% CCZ8/P.P	85	0.0041	–	–	4.39	148.42
15% CCZ8/P.P	106	0.9286	2.0232	0.000470	4.75	340.66
20% CCZ8/P.P	132	1.9419	2.1184	0.001028	4.81	422.48
CCZ8	–	690.7998	3.6628	0.632560	–	–

To investigate the phase and structure of the composite films, the XRD profiles of the synthesized CCZ8 powder and the electrode films as the active material are presented in Figure 3B. The CCZ8 powder presented a distinct broad peak located around 26° that were assigned to the characteristic carbon (002), indicating the presence of long-range ordering in two-dimensional of carbon matrices along with some graphitization (Zhang et al., 2014). PEDOT:PSS film displays a diffraction peak located at around 22.7° assigned to the (200) crystal plane, indicating better crystalline and ordered structure for PEDOT:PSS (Khan et al., 2015). In the PEDOT:PSS electrode films to which CCZ8 are added, both peaks are present but the intensity is decreased due to the increase in the composition of CCZ8 and the decrease in the content of PEDOT:PSS. Raman spectra was conducted under 532 nm semiconductor laser. As shown in Figure 3D, the band around 1430 cm^–1^ is corresponding to the stretching vibration of symmetric Cα = Cβ on the five-member ring of PEDOT (Choi et al., 2010). It indicates that the band slightly moves toward higher wave numbers after the addition of CCZ8 due to the doping effect of carbon nanoparticles (Chiu et al., 2006). In addition, a new band around 1582 cm^–1^ from the tangential tensile mode of high-order pyrolytic graphite could be found in CCZ8/P.P films, indicating the presence of crystalline graphite carbon (Barros et al., 2005; Zhang et al., 2014).

On the other hand, SSA has an important influence on the ions storage of the electrode. Figure 3E characterizes N_2_ adsorption/desorption isotherms and pore size distribution (inset) of 20% CCZ8/P.P electrode film and N_2_ adsorption/desorption isotherms and pore size distribution (inset) of CCZ8 powder also presented in Figure 3F. The pores are expected to improve ion storage capacity and facilitate ion transfer. The SSA and pore size of different addition amounts of the CCZ8/P.P electrode films are summarized in Table 1. As the amount of CCZ8 increases, the surface roughness of the electrode film also increases, and SSA increases from 0.0041 m^2^ g^–1^ to 1.9419 m^2^ g^–1^. However, it is still much smaller than SSA of CCZ8 (690.8 m^2^ g^–1^), primary because the viscous PEDOT:PSS dispersion will block the larger pores and the smaller pores will be covered by it, which could also be seen from the reduction in pore size and total pore volume.

### Fabric Intermediate Layer

#### Morphological Characterization

**FIGURE 4:**
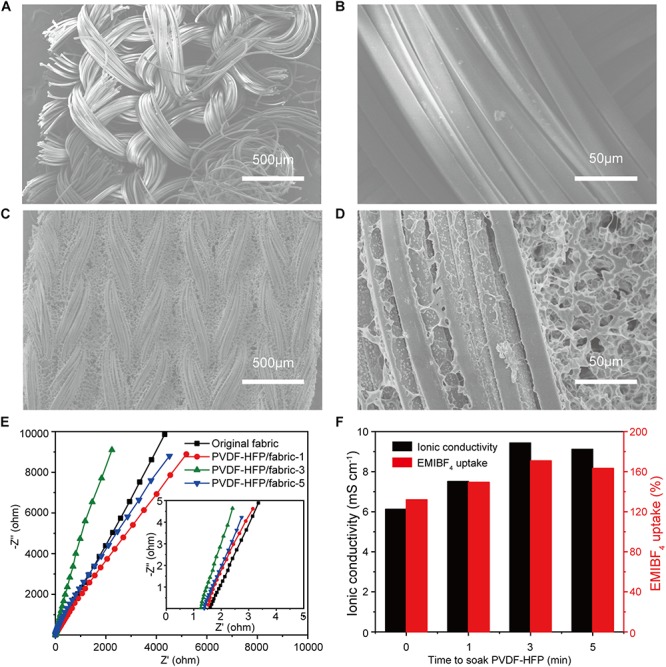
**(A,B)** The surface morphology of polyester fabric at different magnification. **(C,D)** The surface morphology of polyester fabric after immersion in PVDF-HFP at different magnification. **(E)** Nyquist plots of fabric intermediate layer immersed at different times. **(F)** Ionic conductivity and EMIBF_4_ uptake of fabric layer soaked at different times.

In order to characterize the surface morphology of the fabric before and after soaking the PVDF-HFP solution, the woven structure and surface topography of the polyester fabric were presented in Figures 4A,B. It could be clearly seen from Figures 4C,D that the PVDF-HFP layer is adhered to the surface of fabric. The PVDF-HFP layer can store more ions and still have large pores to provide ion migration.

#### Ionic Conductivity

To explore the effect of time to immersion PVDF-HFP on the ionic conductivity of electrolyte layers, the Nyquist plots of electrolyte layer were measured. In this part, after tailored we soak the fabrics the ionic liquid (EMIBF_4_) for 2 h. Figure 4E shows Nyquist plots of fabric intermediate layers immersed for different time and the conductivity is inversely proportional to the impendence. The ionic conductivities of fabric intermediate layers calculated through the equation are shown in Table 2. It could be seen from the table and Figure 4F that the fabric without any treatment could store a certain amount of ions. And the fabric soaked for 3 min PVDF-HFP solution has the best ionic conductivity of 9.43 mS cm^–1^, which is much higher than the values reported in the literature (Liu W. et al., 2017). This is mainly because the PVDF-HFP electrolyte layer prepared by the phase inversion method itself has a porous structure which allows the liquid electrolyte to be embedded in the polymer network, and the ionic liquid is stored in the pores of the polymer network (Liu W. et al., 2017). The increase of ionic conductivity is also related to the high ionic liquid uptake of fabric electrolyte as shown in Figure 4F. The ionic conductivity κ (S cm^–1^) and EMIBF_4_ uptake of fabric electrolyte can be calculated by the following equations: (Kong and Chen, 2014)

**TABLE 2:** Thickness, ionic conductivity and EMIBF_4_ uptake of fabric layers.

**Fabrics**	**Thickness (μm)**	**Ionic conductivity (mS cm^–1^)**	**EMIBF_4_ uptake (%)**
Original fabric	197	6.12	134.52
PVDF-HFP/fabric-1	231	7.51	150.43
PVDF-HFP/fabric-3	239	9.43	171.31
PVDF-HFP/fabric-5	253	9.12	163.52

$$\kappa =\frac{D}{R_{\text{b}}\times \text{S}},I\text{o}nicliquiduptake\left(\%\right)=\frac{\omega_{s}-\omega_{0}}{\omega_{0}}\times 100\%$$

Where D (cm) is the thickness of the fabric electrolyte layer, R_*b*_ (ohm) is the series resistance obtained in the EIS curve, S (cm^2^) is the area where the electrolyte layer is in contact with the stainless steel sheet, ω_0_ and ω_*s*_ represent the mass of the fabric before and after absorbing EMIBF_4_.

### Performances of Textile Actuator

#### Electrochemical Performance

**FIGURE 5:**
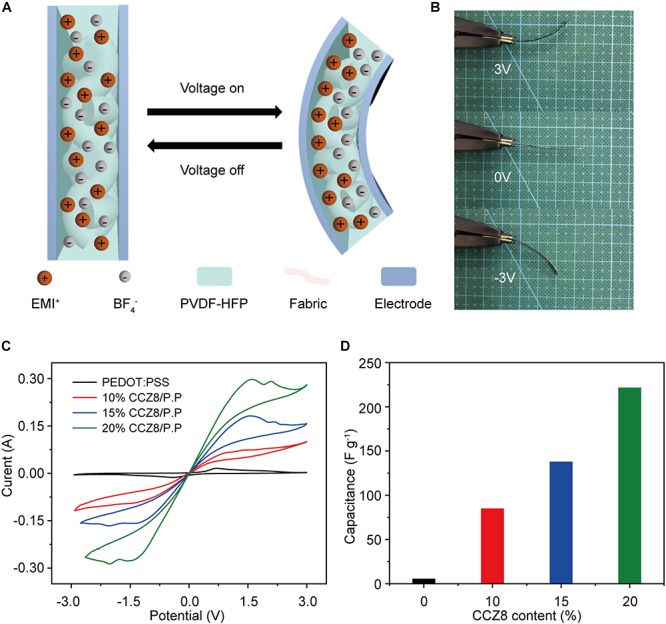
**(A)** Schematic for actuation mechanism of the textile actuator. **(B)** Optical images of the bent actuator under 3 V at 0.1 Hz. **(C)** CV curves for the PEDOT:PSS and CCZ8/P.P electrode based textile actuators at a scan rate of 50 mV s^–1^. **(D)** The calculated capacitances of textile actuators.

In general, there are two mechanisms for i-EAP actuators: one is based on an electric double layer capacitor such as the IPMC actuator; other one is based on a Faraday reaction such as the conductive polymer actuator. In our textile actuator, PEDOT:PSS and CCZ8 are mainly used as Faraday capacitor and electric double layer electrodes, respectively, to achieve bending of the textile actuator device. When a voltage is applied between the two electrode layers, the cations contained in the fabric electrolyte layer move to the cathode layer, and the anions migrate to the anode layer (Figure 5A). The resulting ions movement promotes redox of PEDOT:PSS and an electric double layer on the surface of electrode with a large SSA to achieve swelling and shrinkage of the electrode layer. At low frequencies, the expansion of anode plays a major role in the bending of textile actuator, as opposed to at high frequencies (Terasawa and Asaka, 2016). Optical image of the bent 20% CCZ8/P.P textile actuator under 3 V at 0.1 Hz was presented in Figure 5B. It is obvious that large displacement deflection occurs on both sides of the textile actuator.

In order to evaluate the charge storage behavior of the actuator, cyclic voltammetry (CV) is analyzed at a scan rate of 50 mV/s. The specific capacitance (C) is calculated using the following formula:(Lu et al., 2018)

$$C\,\left(F\,g^{-1}\right)=\frac{S_{A\,r\,e\,a}}{2\,\nu \cdot \Delta \,V\cdot m}$$

Herein, S_*Area*_ = ∫ *I*dV refers to the loop area of the CV curve, ν (V s^–1^) is the scan rate, ΔV (V) is the potential window and m (g) presents the mass of active material in the electrode.

Figure 5C presents CV curves of textile actuators tested at a scan rate of 50 mV. The CV curve of the pristine PEDOT:PSS textile actuator exhibits a typical Faraday capacitance, and the specific capacitance calculated from the integrated area of the CV curve is 5.56 F g^–1^ as shown in Figure 5D. And the calculated special capacitance value of 20% CCZ8/P.P electrode based textile actuator increases to 221.81 F g^–1^, with the electric double layer capacitor mechanisms providing the greatest contribution. It is apparent from the CV curves that the textile actuators based on CCZ8/P.P electrode exhibit higher electric double layer capacitance, ascribing the electrode film with CCZ8 added having larger SSA. Another reason for the increase in capacitance is that the conductivity of the CCZ8/P.P electrode is higher than that of the pristine PEDOT:PSS electrode.

#### Actuation Performance

The mechanical bending deformation test of the textile actuators with the same dimension (0.5 cm × 3 cm size) was performed by multi-potential step. The strain difference (ε), strain rate (ε_*r*_), stress (σ), stress rate (σ_*r*_), energy density (E), and power density (P) of the textile actuator are calculated by the following equations:(Wang et al., 2017; Wu et al., 2019)

$$\varepsilon =\frac{2\,\delta \,d}{\delta^{2}+L^{2}}\,\varepsilon_{r}=4\,\varepsilon \,f;\sigma =\varepsilon \,Y;\sigma_{r}=\varepsilon_{r}\,Y;E=\frac{Y\,\varepsilon^{2}}{2};P=2\,\varepsilon \,f\,Y$$

Here, δ (mm), d (mm), L (mm), f (Hz), and Y (Pa) refer to half of the peak-to-peak displacement, the thickness, free length, frequency, and Young’s modulus of textile actuator, respectively.

**FIGURE 6:**
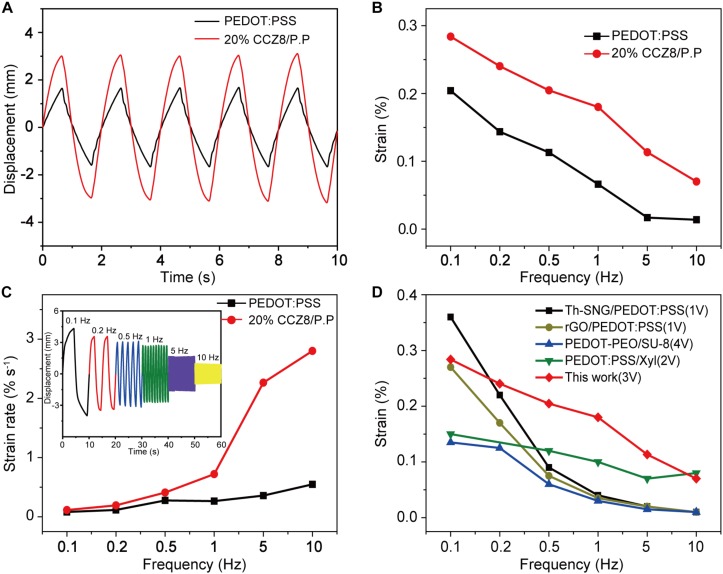
Bending actuation performances of PEDOT:PSS and 20% CCZ8/P.P textile actuator. **(A)** Peak to peak displacement under 3 V at 0.5 Hz. **(B)** Strain difference under 3 V from 0.1 Hz to 10 Hz. **(C)** Strain rate under 3 V from 0.1 Hz to 10 Hz. Inset: displacements of 20% CCZ8/P.P textile actuator with increasing frequencies. **(D)** Comparison of bending strain difference–frequency curves of the reported PEDOT:PSS based actuators dealing with low-voltage operation.

The textile actuators are largely bent according to the electrical stimulus. Peak to peak displacement of the 20% CCZ8/P.P actuator is significantly larger than that of pristine PEDOT:PSS, and the response of the displacement to voltage is consistent and rapid (Figure 6A). Figures 6B,C display the actuation performance of textiles actuator devices with wide frequencies (0.1–10 Hz). At all frequencies, the 20% CCZ8/P.P textile actuator has higher strain difference and strain rate than that of the PEDOT:PSS textile actuator, thanks to the electrode layer with CCZ8 added possessing higher conductivity and SSA. In particular, the strain difference of the 20% CCZ8/P.P textiles actuator at 0.1 Hz is 0.28% higher than that of PEDOT:PSS textile actuator (0.20%). More importantly, the 20% CCZ8/P.P textile actuator still remains an actuation strain difference of 0.07% at 10 Hz, while the PEDOT:PSS textile actuator is only 0.01%. The 20% CCZ8/P.P textile actuator generates a maximum strain rate of 2.8% s^–1^ exceeding that of the PEDOT:PSS textile actuator at only 0.55% s^–1^. In the inset of Figure 6C, the displacement decreases as the frequency increases, primarily because the ions do not have sufficient time to transfer to the electrodes at high frequencies (Kotal et al., 2016). As expected, compared to the other PEDOT:PSS based actuators reported in the literature, the 20% CCZ8/P.P textile actuator dealing with low-voltage operations under different input frequencies shows better bending strain as depicted in Figure 6D (Li et al., 2014; Kotal et al., 2016; Maziz et al., 2016).

**FIGURE 7:**
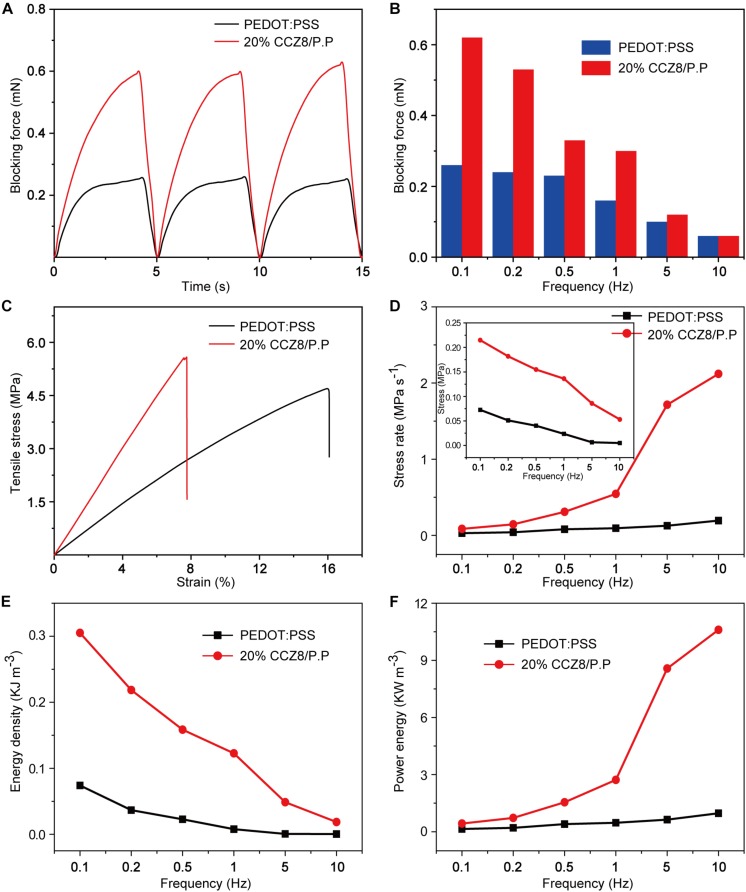
Actuation performances of PEDOT:PSS and 20% CCZ8/P.P textile actuator. **(A)** Time-dependent blocking forces at 0.1 Hz. **(B)** Frequency-dependent blocking forces. **(C)** The tensile stress-strain curves. **(D)** Frequency-dependent stress rates. Inset: frequency-dependent stress. **(E)** Frequency-dependent energy densities. **(F)** Frequency-dependent power densities.

Apart from large strain and fast response, textiles actuators also exhibit large blocking forces. The blocking forces of all textile actuators were measured under 3 V at different frequency from 0.1 to 10 Hz. As shown in Figure 7A, the 20% CCZ8/P.P textile actuator generates the blocking force of 0.62 mN under the applied voltage of 3 V at 0.1 Hz, which is much higher than that of the PEDOT:PSS textile actuator (0.26 mN) and the conducting polymer actuator based on PEDOT:PSS/MWCNTs composite electrode (0.54 mN) (Wang et al., 2017). In addition, owing to insufficient time for ions diffusion at high frequencies mentioned before, the blocking force of the textile actuator gradually decreases as the frequency increases (Figure 7B). The textile actuator generates such a large blocking force because of its high stiffness. Figure 7C shows that the 20% CCZ8/P.P textile actuator exhibits maximum tensile strength and elongation of 5.59 MPa and 7.80%, respectively. Meanwhile, the Young’s modulus of the 20% CCZ8/P.P textile actuator reaches 75.69 MPa, which is 113% enhancement than that of the PEDOT:PSS textile actuator (35.54 MPa).

To gain insight into the actuation performance of textile actuators, stress and stress rates were investigated (Figure 7D). The 20% CCZ8/P.P textiles actuator generates a stress rate of 2.12 MPa s^–1^ at 10 Hz, extremely higher than that of the PEDOT:PSS textile actuator (0.19 MPa s^–1^). More importantly, the 20% CCZ8/P.P textile actuator displays a maximum actuation stress of 0.21 MPa at 0.1 Hz, which is approaching to that of human skeletal muscle (0.3 MPa) (Baughman, 2005). Moreover, as Figures 7E,F presented, high energy density and power density of the 20% CCZ8/P.P textile actuator reach 0.31 KJ m^–3^ and 10.60 KW m^–3^ exceeding that of the PEDOT:PSS textile actuator (0.07 KJ m^–3^ and 0.97 KW m^–3^), respectively. There is no doubt that composite electrodes with CCZ8 added have larger SSA and conductivity for the migration and accumulation of ions into electrode compared to pristine PEDOT:PSS electrode, resulting in better actuation performance of textile actuators.

**FIGURE 8:**
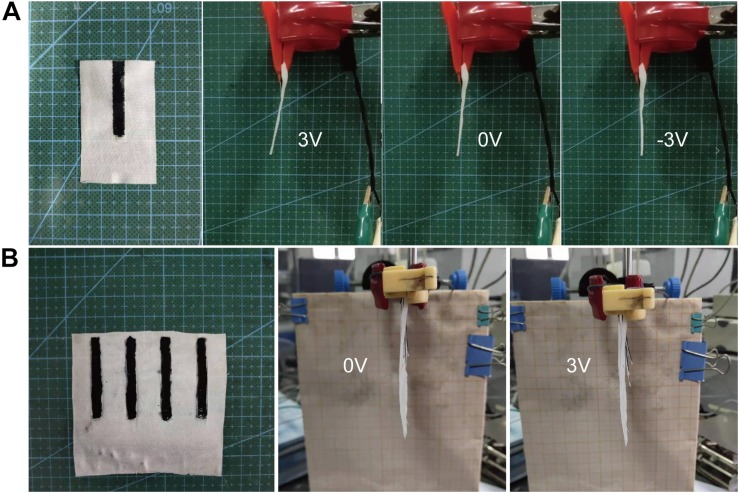
Applications of textile actuators. **(A)** Small electrode textile actuator work at 0.1 Hz (the fabric area is 10 times that of the electrode). **(B)** Several parallel devices under the applied voltage of DC 3 V.

To determine the potential applications based on the large actuation deformation, we focus on the proof of concept in wearable filed developed by electrochemical textile actuators. Based on excellent output and bending motion, the textile actuator can drive large fabric (3 cm × 5 cm size) with smaller electrodes (0.5 cm × 3 cm size) as Figure 8A shows. The textile actuator can still produce a relatively significant displacement under the operating voltage of 3 V at 0.1 Hz, which opens up possibilities for the application of the actuator device to wearable robots and auxiliary medical fields. In addition, as shown in Figure 8B, an array of four textile actuator devices connected in parallel can achieve large deflection under DC 3 V. Each device in the array is deflected relative to the fabric substrate. When the ambient temperature changes, the porosity of the entire textile can be changed by adjusting the bending degree of the actuator, thereby increasing or decreasing their thermal insulation value. The temperature adjustment of the garment shows straightforward evidence for great potential in smart clothing applications. Furthermore, because ionic liquid (EMIBF_4_) is toxic and has certain hazards to the human body, the textile actuators will be appropriately packaged according to different application scenarios.

## Discussion

As an important component of composite electrode materials, the carbonized MOF skeleton is synthesized by high temperature calcination of CNTs/ZIF-8 in a tube furnace (Lu et al., 2017). The porous continuous framework prepared by this simple synthetic route provides a path for the electrons to move and diffuse as a filler and provides a larger SSA for the ions storage. In order to adjust the viscosity of the electrode solution conveniently, we used PEDOT:PSS pellets instead of dispersion in this experiment. In the printing process, if the viscosity of the electrode solution is too high, the composition distribution is not uniform, and if the viscosity is too low, the solution will flow out from the substrate. Furthermore, we prepare composite electrodes by a direct mixing method which is efficient, convenient, suitable for large-scale preparation. Our routing scheme for conducting polymer electrode with better performance provides a reference for the study of other flexible electrodes.

Since the general textiles have large pores and a certain hydrophilicity, the electrode solution cannot directly form a film on the surface of the pristine textile. To solve this problem, hydrophobic PVDF-HFP solution is prepared to soak the textile. We have found that this method not only prevents the electrode solution from penetrating the fabric, but also increases the ionic conductivity of the fabric intermediate layer. However, electrode solution using water as solvent still does not form film on the surface of the hydrophobic PVDF-HFP. We etch hydrophobic textiles with nitrogen plasma to modify the surface for obtaining PVDF-HFP with a certain hydrophilicity. Finally, the resulting fabric layer of has a high ionic conductivity and a tight interface with the electrode material. And this kind of fabric as an intermediate layer has great application prospects in the field of wearable electronic devices.

The final textile actuator combines textiles with electronics and possesses superior actuation performance. The textile device can operate at low voltages of 3 V due to the lower redox potential of the conductive polymer and produce large strain and blocking force. In addition, textile actuator has higher energy density and lower energy consumption thanks to the large SSA of the electrodes. Because of the chemical stability of PEDOT:PSS, the textile actuator can work stably in the air for a long time and operate at wide frequencies. Because ionic liquids are toxic and have certain hazards to the human body, they will be appropriately packaged according to different applications. PEDOT:PSS is almost non-toxic and does not cause discomfort when it is in close contact with the body, therefore it is predictable that textile actuator will receive more attention in applications such as wearable and paramedical.

## Conclusion

In summary, a high-performance textile actuator based on PEDOT:PSS/CCZ8 composite electrodes was reported. The porous structure of CCZ8 endows large SSA for the electrode material, which facilitate ion transfer and accumulation under actuation process. High conductivity and excellent mechanical properties of the electrode material also play key roles in improving actuation performance. In addition, we treated the fabric by etching with nitrogen plasma after immersing the PVDF-HFP solution. The obtained fabric intermediate layer not only shows high ionic conductivity (9.43 mS cm^–1^), but also prevents short circuits of the device. Finally, we combined the fabric with the electrode material by direct dipping to prepare textile actuators. It was found that the 20% CCZ8/P.P textile actuator performers higher tensile strength (75.69 MPa) and large special capacitance (221.81 F g^–1^). Furthermore, 20% CCZ8/P.P electrode based textile actuator is capable of producing large actuation strain difference (0.28%), stress (0.21 MPa) under 3 V at 0.1 Hz, and high strain rate (2.80% s^–1^), stress rate (2.12 MPa s^–1^) at 10 Hz. Furthermore, large blocking force (0.62 mN) and high energy density (0.31 KJ m^–3^) at 0.1 Hz were obtained for the textile actuators. By exploiting these outstanding features, we anticipate that conducting polymer/MOF-derivative composite electrode and the textile actuator design will advance new generation electrode architecture as well as guide substantial progress in the fields of wearable, auxiliary medicine, and bionic robot.

## Data Availability Statement

All datasets generated for this study are included in the article/supplementary material.

## Author Contributions

WC conceived the idea. WC and YW designed the research and devices. YW, YY, and CL collected and analyzed the data. YW, YY, CL, and YL prepared the manuscript. All authors revised the final manuscript.

## Conflict of Interest

The authors declare that the research was conducted in the absence of any commercial or financial relationships that could be construed as a potential conflict of interest.
